# AI and Primary Care: Scoping Review

**DOI:** 10.2196/65950

**Published:** 2025-08-15

**Authors:** Gellert Katonai, Nora Arvai, Bertalan Mesko

**Affiliations:** 1 Kálmán Laki Doctoral School of Biomedical and Clinical Sciences, University of Debrecen, Egyetem tér 1, Főépület földszint 15/A, Debrecen, 4032, Hungary, 36 52-258-010 ext. 58010; 2 Department of Family Medicine, Semmelweis University, Budapest, Hungary; 3 The Medical Futurist Institute, Budapest, Hungary; 4 Meducation Hungary Kft, Budapest, Hungary

**Keywords:** artificial intelligence, primary care, general practice, AI, scoping review, primary healthcare, thematic analysis, drug management, disease screening, AI integration, integration

## Abstract

**Background:**

Primary health care (PHC) is critical for delivering accessible and continuous care but faces persistent challenges such as workforce shortages, administrative burden, and rising multimorbidity. Artificial intelligence (AI) has the potential to support PHC by enhancing diagnosis, workflow efficiency, and clinical decision-making. However, existing research often overlooks how AI tools function within the complex realities of primary care and how clinicians and patients experience them.

**Objective:**

This scoping review maps the landscape of AI applications in PHC, with a focus on empirical studies involving direct engagement from PHC stakeholders. The review emphasizes real-world settings, clinical workflows, and the alignment of AI tools with the values and complexity of generalist care.

**Methods:**

Following Joanna Briggs Institute methodology and PRISMA-ScR (Preferred Reporting Items for Systematic Reviews and Meta-Analyses Extension for Scoping Reviews) guidelines, we searched PubMed, Web of Science, and Scopus databases up to April 13, 2024. Inclusion criteria were empirical, peer-reviewed studies published in English between January 2010 and April 2024, involving direct stakeholder interaction (general practitioners, nurses, or patients) in real-world PHC settings, evaluating AI applications (eg, diagnostics, workflow optimization, and documentation). Exclusions included algorithm-only validations, pediatric populations, secondary or tertiary care contexts not explicitly addressing PHC workflows, nonempirical research (eg, editorials or protocols), and non-English studies. We used thematic analysis to synthesize findings related to study aims, AI applications, and stakeholder roles.

**Results:**

Of 5224 identified records, 73 studies met the inclusion criteria. Studies were grouped into four main themes: (1) early intervention and decision support (n=21; 29%), (2) chronic disease management (n=16; 22%), (3) operations and patient management (n=12; 16%), and (4) acceptance and implementation experiences (n=24; 33%). AI tools frequently demonstrated strong technical accuracy, particularly in diagnostic decision support. However, implementation in routine practice was often limited by usability barriers, workflow misalignment, trust concerns, equity gaps, and financial constraints.

**Conclusions:**

Overall, AI holds significant potential to support PHC, especially when aligned with clinical reasoning, workflow needs, and relational care models. However, persistent implementation barriers such as usability challenges, training gaps, and workflow integration issues must be addressed. The evidence included in this review is limited by heterogeneity in study design and the predominance of small-scale feasibility studies. Future research should prioritize pragmatic trials, co-design with PHC professionals, and anticipatory planning using future methods to ensure responsible and equitable implementation.

## Introduction

Primary health care (PHC) is the foundation of equitable, accessible, and continuous health service delivery across populations. As the first point of contact in the health system, PHC manages undifferentiated symptoms, provides preventive services, and coordinates chronic disease care. In many countries, general practitioners (GPs) deliver PHC through the family medicine model, which emphasizes continuity, comprehensiveness, and long-term therapeutic relationships [1]. However, it is increasingly challenged by workforce shortages, administrative tasks, and clinician burnout [2-4]. These issues are intensified by aging populations, multimorbidity, and persistent health inequalities, creating an urgent need for new strategies to maintain high-quality, person-centered care [4,5].

Digital technologies have become integral to primary care delivery as part of efforts to improve coordination, reduce administrative workload, and support clinical decision-making. Among these innovations, artificial intelligence (AI) has emerged as a particularly influential development, with applications spanning diagnostics, workflow optimization, and documentation [1,6-12]. As the field shifts from narrow, task-specific models to more flexible, multimodal, and generative approaches, it is becoming increasingly important to evaluate how these systems interact with everyday practice [13].

Despite growing interest, the literature on AI in PHC remains fragmented. Many studies focus on specific tasks, such as risk prediction or documentation support [14-16]. Others examine where and by whom AI tools are developed, often highlighting the dominance of bioinformatics and the limited involvement of frontline clinicians [17]. Previous reviews have typically categorized AI tools by technical function or task type but have rarely examined how these tools are implemented in clinical PHC or how they support PHC values such as continuity, accessibility, and patient engagement [18]. With the rise of more adaptable AI systems, particularly generative models, a systematic evaluation is therefore warranted at this stage of development [13].

This scoping review identifies empirical studies on AI in PHC that involve direct participation of key stakeholders, including health care providers such as GPs and nurses, as well as patients. By focusing on real-world use, workflow integration, and clinical relevance, the review offers a practice-oriented overview of current applications and highlights areas for future research and implementation.

## Methods

The review was conducted following the Joanna Briggs Institute methodology for scoping reviews and is reported per the PRISMA-ScR (Preferred Reporting Items for Systematic Reviews and Meta-Analyses Extension for Scoping Reviews) guidelines [19,20]. The completed PRISMA-ScR checklist is provided in Checklist 1. Eligibility criteria were developed using the Population, Concept, Context framework to ensure methodological rigor [21]. Detailed inclusion and exclusion criteria are outlined in Table 1.

**Table 1.** Eligibility criteria used in the scoping review.^a^

Domain	Inclusion criteria	Exclusion criteria
Population	PHC^b^ stakeholders directly involved with AI^c^ (GPs^d^, nurses, other PHC clinicians, or patients)	Studies with no stakeholder interaction (such as algorithm-only validation) or pediatric patients
Concept	AI applications tested in practice (diagnostics, workflow, triage, documentation, etc)	Digital tools without explicit AI components or medical education usage
Context	Real-world PHC settings (community clinics or GP offices)	Secondary or tertiary care, unless explicitly addressing PHC workflows
Study design	Empirical peer-reviewed research	Editorials, reviews, protocols, and conference abstracts
Language	English	Non-English
Date range	January 01, 2010 to April 13, 2024	Outside date range

a Alternate text: studies were included if they involved empirical, peer-reviewed research published in English between January 1, 2010, and April 16, 2024. Eligible studies focused on artificial intelligence applications implemented or tested in real-world primary health care settings, involving direct interaction with primary health care stakeholders (eg, general practitioners, nurses, or patients). Studies were excluded if they lacked stakeholder interaction (eg, algorithm-only validations), focused solely on pediatric populations, or were conducted exclusively in secondary or tertiary care contexts without relevance to primary health care workflows. Additional exclusions applied to nonempirical work (eg, editorials or protocols) and non-English publications.

b PHC: primary health care.

c AI: artificial intelligence.

d GP: general practitioner.

A 2-step search strategy was conducted per recommended guidelines [19]. The complete search strategy, including database-specific queries, is provided in Multimedia Appendix 1. First, a preliminary search was performed in PubMed by author GK to identify relevant keywords and indexing terms. Based on these findings, a comprehensive search was then conducted across PubMed, Web of Science, and Scopus, using a combination of controlled vocabulary (eg, MeSH, Medical Subject Headings terms) and free-text keywords related to AI and PHC, applied with Boolean operators. Identified studies were exported to Mendeley (version 1.109.1; Elsevier) and shared among the authors for further screening.

After the search, duplicates were removed. Title and abstract screening were independently conducted by GK and BM, who assessed each study against the inclusion criteria. Studies deemed potentially relevant proceeded to the full-text review phase, where both reviewers conducted a detailed evaluation.

Screening was carried out in multiple rounds, with iterative discussions to resolve uncertainties or discrepancies. Disagreements were resolved by consensus, with GK acting as the final reviewer. Additionally, NA conducted a final scan of the included studies to ensure consistency and alignment with the eligibility criteria.

Relevant data from the included studies were extracted and aggregated in Microsoft Excel (version 2402; Microsoft Corp). The extraction included key study characteristics: title, authors, year, journal, DOI, study design, setting or context, population or participants, data sources, clinical setting, key findings, summary, and the thematic group. The full data extraction table sorted by themes is provided in Multimedia Appendix 2.

To further structure the evaluation, emerging themes were identified through the analysis of study aims, AI applications, and stakeholder roles, facilitating a structured mapping of evidence gaps and trends [22]. Following an initial familiarization with the dataset, open coding was conducted manually within the generated spreadsheet. Codes were iteratively reviewed and grouped into potential themes by all 3 researchers, then refined through multiple rounds of web-based and in-person discussions. The final themes were determined based on their recurrence across studies and their relevance to the research question. These themes informed the final synthesis, providing a structured lens for evaluating the included studies.

## Results

### Overview

**Figure 1.**
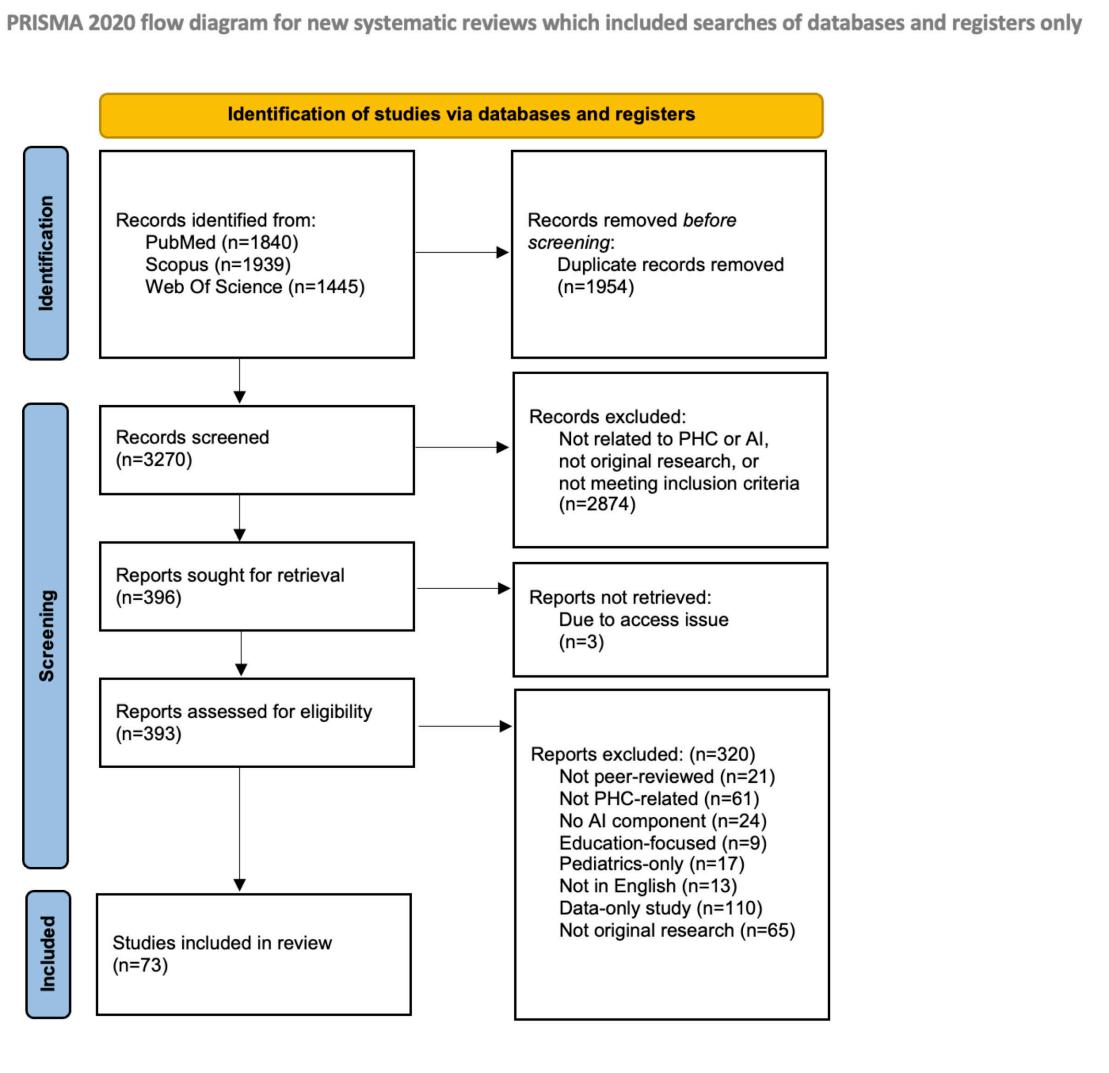
PRISMA 2020 flow diagram showing this study’s selection process for the scoping review. Alternate text: The review included peer-reviewed empirical studies published between 2010 and 2023, focusing on the implementation or evaluation of AI tools in PHC settings. A total of 5224 records were identified from PubMed, Scopus, and Web of Science. After removing 1954 duplicates, 3270 records were screened by title and abstract. Of these, 2874 were excluded for not meeting eligibility criteria, such as lack of PHC or AI relevance, or not being original research. Full texts were assessed for 396 records, of which 3 could not be retrieved and 320 were excluded. The final review included 73 studies, representing adult and general populations across diverse global settings, with applications targeting diagnostic support, triage, and decision-making tasks in PHC. AI: artificial intelligence; PHC: primary health care; PRISMA: Preferred Reporting Items for Systematic Reviews and Meta-Analyses.

We identified 5224 records, with 1954 duplicates removed. After screening 3270 titles and abstracts, 2874 studies were excluded. A total of 396 papers were assessed in full text. Three full texts were inaccessible, and 320 were excluded based on eligibility criteria, resulting in 73 studies included in the final review (Figure 1).

These 73 studies encompassed diverse study designs and methodological approaches. The majority used quantitative research designs, including diagnostic accuracy studies, validation studies, and retrospective cohort analyses. A smaller subset used mixed-methods approaches, integrating quantitative performance assessments with qualitative evaluations of AI implementation. Additionally, 2 studies applied Delphi consensus methodology or choice experiments to understand expert and stakeholder perspectives on AI in clinical workflows.

The studies were geographically diverse, with a substantial number conducted in the United Kingdom, Germany, France, and North America, alongside contributions from other European, Asian, and Australian health care systems. Data sources varied widely, ranging from electronic health records (EHRs) and telemedicine platforms to AI-powered decision support systems and digital consultation transcripts.

We conducted a thematic analysis in which each study was assigned to 1 of 4 primary themes. Of the 73 studies included, 21 explored early intervention and decision support, 16 examined comprehensive chronic disease management and coordinated care, 12 addressed primary care operations and patient management, and 24 focused on acceptance, implementation, and experiences of AI in primary care. The distribution of these themes is illustrated in Table 2.

**Table 2.** Thematic classification of 73 studies included in the scoping review.^a^

Theme	Studies (N=73), n (%)	
Early intervention and decision support	21 (29)	
Chronic disease management	16 (22)	
Operations and patient management	12 (16)	
Acceptance and implementation	24 (33)	

a Studies were thematically categorized based on their primary focus using an inductive thematic analysis. Of the 73 peer-reviewed empirical studies, 21 (28.8%) addressed early intervention and clinical decision support; 16 (21.9%) focused on chronic disease management and coordinated care pathways; 12 (16.4%) explored primary care operations and patient management, including workflow optimization; and 24 (32.9%) examined the acceptance, implementation, and lived experiences of artificial intelligence integration in primary health care.

### Theme 1: Early Intervention and Decision Support

Several studies evaluated AI for earlier detection of cancer and cardiovascular conditions. One model using patient records predicted colorectal cancer with 73% sensitivity and 84% specificity, supporting earlier diagnostic decision-making [23]. An AI tool using routine blood-test data predicted risk more accurately than a statistical model, with performance scores of 86% and 80%, respectively [24]. Cardiovascular risk detection with an AI-interpreted electrocardiogram (ECG) program raised low-ejection-fraction heart failure diagnoses from 1.6% to 2.1% [25], and a follow-up analysis found that frequent tool users were twice as likely to detect the condition [26]. A combined ECG-stethoscope with an AI algorithm identified reduced ejection fraction with 92% sensitivity and 80% specificity [27], while the Conformité Européenne–certified PMcardio (Powerful Medical, Inc) app detected atrial fibrillation with 97% sensitivity and 99% specificity in the doctor’s room [28].

AI also shows promise for skin lesion assessment in primary care: an AI morphology classifier reached 68% on top-1 accuracy across 44 conditions [29], and a handheld elastic-scattering spectroscopy device boosted skin-cancer diagnostic sensitivity from 67% to 88% [30]. Teledermatology research shows that AI assistance cut biopsy and referral rates while increasing clinician-dermatologist agreement from roughly 48% to 58% across 1048 cases [31], a prospective decision-support tool for melanoma screening achieved a 99.5% negative-predictive value in 253 lesions [32]. A feasibility pilot showed 90% sensitivity and 65% specificity for AI-assisted melanoma detection with high usability [33]. Four further studies reported accuracies ranging from 39% to 89%, often with sensitivities above 90% [34-37].

In ophthalmology, machine-learning classifiers for glaucoma referral achieved up to 60% sensitivity and 77% specificity [38], while an AI-assisted telemedicine platform detected urgent retinal disease with 97% sensitivity and 99% specificity, and cut workload by 96% [39]. Beyond disease-specific applications, machine learning systems are demonstrating superior performance in general diagnostic tasks within primary care: a text-note classifier identified primary headache disorders with 85% accuracy versus 66% for GPs [40], while 1 ensemble AI model identified significant liver fibrosis with 94% overall accuracy and a 98% negative predictive value, performing better than standard blood-based scoring methods [41]. AI-driven decision aids can also enhance prescribing: 1 urinary-tract-infection management tool boosted treatment success from 75% to 84% across 36 practices [42], while another study on acute respiratory infections reported 39%-77% uptake of an antibiotic-prescribing aid, potentially reducing unnecessary antibiotic use [43].

Overall, most tools identified in the review targeted highly relevant conditions such as cancer, cardiovascular disease, and retinal disorders, where early diagnosis is especially impactful. These tools showed high diagnostic accuracy and were often based on structured clinical data sources such as ECGs, dermoscopic images, and EHRs. Key enablers included diagnostic accuracy, alignment with existing workflows, and support for timely decision-making without undermining clinical autonomy.

### Theme 2: Comprehensive Chronic Disease Management and Coordinated Care

An AI-driven system for classifying digital specialist communication messages categorized them correctly in 86% of cases while requiring only 10% of the labeled data [44]. Machine learning also supports chronic care in PHC: a decision support system integrating GP engagement and EHR data improved diabetes management by increasing complication-free rates by up to 12% [45], while an AI-based diabetes program in Mexico achieved a 5% improvement in glycemic control, identifying subgroups that benefited most from GP-led interventions [46].

Researchers have evaluated a range of AI systems for diabetic retinopathy screening: deep-learning classifiers, combined macular degeneration detection models, teleplatforms with pupil dilation, automated graders, and handheld devices. These systems achieved sensitivities of 87%-100% and specificities of 89%-98% [47-52]. Implementation studies for retinopathy screening have examined real-world uptake, workflow impact, and patient follow-up. A telemedical form engaged 85% of clinicians [53], and a real-world AI grading software increased on-time report completion by 12 percentage points but showed only moderate concordance with endocrinologists. In low-resource AI screening, 100% sensitivity was maintained and follow-up adherence doubled [54,55].

AI can also support medication management in PHC: an AI web application reduced drug-interaction detection time from 37 minutes to 33.8 seconds, detecting 75.3% of potentially inappropriate medications [56], while a CDSS for polymedicated older adult patients improved prescribing safety and reduced adverse‐drug events in feasibility testing [57]. AI also aids respiratory and speech disorders: a vocal-cord pathology classifier achieved an *F*_1_-score of 0.98 (which means near-perfect balance of sensitivity and precision), outperforming specialist review in dysphonia detection [58], while qualitative research on AI-supported spirometry highlighted the need for robust validation and specialist integration [59].

Across studies, AI supported chronic disease management by enabling earlier risk stratification, safer prescribing, and more consistent follow-up. Tools were most effective when they were embedded in existing care processes, drew on longitudinal data, and supported GP-led coordination. Rather than replacing clinical workflows, these systems helped structure care across time, improving communication, safety, and responsiveness for patients with complex needs.

### Theme 3: Primary Care Operations and Patient Management

An AI model trained on 239 GP consultation recordings assigned clinical codes with approximately 50% accuracy, indicating potential for partially automating routine coding tasks [60]. Another AI approach accurately flagged 98% of consultations suitable for remote management, although it correctly identified the specific reason for the consultation, such as prescription renewals versus new symptoms, in only 48% of cases [61]. One triage AI tool matched physician assessments in only 17% of cases overall, though it performed substantially better when identifying nonurgent (74%) compared to urgent cases (42%) [62]. A different respiratory triage model accurately excluded pneumonia in low-risk patients, reducing unnecessary chest x-ray referrals by 25% [63].

AI has been explored to streamline documentation and workflows. Ambient voice technology that automatically captures clinical conversations decreased documentation time by 28.8%, alleviating physician burnout [64]. ML-based audits of EHRs identified 80% of GP-assessed heart failure cases and reduced screening workloads by 33%, illustrating AI’s utility in medical record analysis [65]. Natural language processing models examining EHR notes identified discussions of prediabetes with high sensitivity (0.98) and specificity (0.96), revealing opportunities to address care gaps through early interventions [66].

An AI-based risk prediction algorithm detected 45,493 new atrial fibrillation cases at £3994 (US $5423) for each additional year of healthy life gained, demonstrating cost-effectiveness [67]. Budget modeling indicates that a wider rollout could cut undiagnosed atrial fibrillation by 27%, prevent 3299 strokes, and reduce health care costs [68]. A machine-learning–based decision-tree model revealed that GPs based lipid-lowering prescriptions on individual risk factors and sociodemographic profiles rather than on guideline-recommended absolute-risk thresholds [69]. Appointment no-show predictors achieved 47% sensitivity and 79% specificity, enabling targeted reminders and fewer missed visits [70]. When primary care physicians evaluated chart summaries generated by topic models, they rated the 100-topic version as more coherent and appropriately detailed than 50 or 150-topic models, demonstrating its superior interpretability [71].

Taken together, these studies show that AI is increasingly being tested out to support primary care operations, including triage, documentation, coding, and scheduling. While tools vary in performance, many have demonstrated meaningful improvements in efficiency, diagnostic support, and administrative workload reduction. AI tools that addressed operational tasks were most effective when they reduced clinician burden without compromising clinical autonomy. Tools were most effective when reducing clinician burden without compromising autonomy, particularly when integrated with EHRs, designed for interpretability, and applied to low-complexity tasks.

### Theme 4: Acceptance, Implementation, and Experiences of AI in Primary Care

Physician attitudes, patient perspectives, usability, and system factors shape AI integration. One mixed-methods study identified optimism and perceived innovativeness as key predictors of acceptance, while privacy concerns and health awareness influenced readiness [72]. A survey of GPs emphasized priorities such as urgent diagnoses, integration with EHRs, and personalized care, though concerns about clinical autonomy and tool usability remained [73]. In a discrete choice experiment, primary care providers preferred AI for breast cancer screening as a triage support system rather than a standalone diagnostic solution [74].

Stakeholders and professionals across multiple contexts highlighted factors influencing AI adoption. Younger physicians were generally more open to AI, though privacy and regulatory concerns remained a barrier [75]. Risk profiling and administrative support emerged as top priorities, but equity and data quality issues limited broader implementation [76]. Financial, technical, and attitudinal challenges were frequently cited in studies of AI-based diabetic retinopathy screening [77], with cost, reimbursement, and usability ranked as key enablers of GP engagement [78]. Qualitative work further emphasized the gap between envisioned AI use and practical realities, underscoring the need for co-creation, high-quality data, and ethical safeguards [79]. Among professionals, 85.7% reported understanding AI and 91.4% expressed interest in training, though concerns about ethics and interoperability remained [80].

Physician trust and system readiness also impact adoption. Interview-based research found that GPs’ concerns about autonomy and trust hindered AI uptake [81], and deliberative dialogues emphasized bias, regulation, and co-design as critical for implementation [82]. Surveys on AI for nonmelanoma skin cancer reported enthusiasm for diagnostic support, but cost and software availability limited broader use [83]. Perspectives on AI-assisted skin cancer detection pointed to benefits in diagnostic accuracy and care pathways, yet highlighted bias, usability, and shifting professional roles as key concerns [84]. A Delphi consensus called for rigorous design, evaluation, and ethical safeguards, noting challenges with integration and workflow [85].

Patient attitudes and broader system challenges further shape AI adoption. One qualitative study found that while patients supported AI for decision support, they emphasized the importance of maintaining GP autonomy and trust, particularly when sharing personal data [86]. Observational research on AI-enabled diabetic retinopathy screening reported improved access and uptake, with patients expressing willingness to continue screenings in general practice despite some implementation challenges [87]. A feasibility study on AI-based symptom checkers during the pandemic found that nearly half of patients considered them useful, though physicians raised concerns about usability and integration into clinical workflows [88]. In a pilot conducted in a GP waiting room, most patients, especially younger users, found an AI-driven symptom checker helpful for initial self-assessment [89].

A stakeholder-informed agenda prioritized AI for documentation, triage, and decision support, with emphasis on equity, safety, and training [90]. Workflow analyses emphasized user-centered design, system interoperability, and communication integration as key requirements for AI decision support tools [91]. GPs expressed support for doctor-AI collaboration but raised concerns about usability and workflow integration [92]. A mixed-methods study identified equity, workflow, and technical challenges as key barriers to AI implementation [93]. A survey found that GPs with higher self-efficacy tended to view AI more positively [94]. Family physicians reported low levels of AI-related anxiety and indicated that AI-specific training could support integration [95].

Taken together, the studies indicate that successful AI integration in primary care depends on clinician trust, perceived usefulness, and alignment with clinical roles. Adoption was influenced by usability, data quality, ethical transparency, and regulatory readiness. Key enablers included user-centered design, structured training, and cocreation with stakeholders. Barriers are commonly related to interoperability and unclear clinical value. Across studies, PHC professionals were most often engaged through post hoc feedback or during tool testing, with fewer examples of involvement in the design or validation phases. Across studies, implementation success depended on addressing both technical performance and professional integration needs.

## Discussion

### Principal Findings

This scoping review identified a wide range of AI applications in primary care, with studies grouped around 4 thematic areas: early diagnosis, chronic disease management, operational support, and implementation experiences. Many tools demonstrated strong technical performance, though most are in the early implementation stage and are not yet integrated into routine workflows. Across themes, studies frequently identified recurring enablers and challenges, including workflow alignment, clinician trust, and training availability. These findings suggest that technical accuracy alone is not sufficient to ensure real-world adoption in primary care.

### Interpretation of Findings

Several recurring patterns emerged across the included studies. The consistent performance of structured-data-based tools suggests that aligning AI inputs with standardized clinical formats may be critical for diagnostic reliability and system integration in PHC. Tools that were designed to fit within routine clinical workflows, such as those used for screening, prescribing, or documentation, tended to be more usable and were adopted more readily, particularly when they reduced administrative burden while preserving clinician autonomy. In many cases, implementation success depended more on human and organizational factors than on technical capability. These included clinician trust, perceived usefulness, availability of training, and compatibility with existing professional roles. However, few studies engaged PHC professionals during the development phase, and most reported only postimplementation feedback, limiting opportunities for early alignment with clinical needs. Patient involvement was rare and typically limited to user testing or acceptability assessments. Together, these findings suggest that effective AI tools in primary care must respond to the relational, interpretive, and operational aspects of general practice. While these design features were present in several tools, broader integration was often limited by structural constraints that are explored in the following sections.

### Technical Potential Versus Real-World Constraints

The reviewed studies demonstrate AI’s potential to enhance clinical decision-making, risk stratification, and operational efficiency. Despite promising technical performance during early pilot testing, most AI tools for PHC remain at the proof-of-concept stage, with limited integration into clinical workflows and unclear real-world impact. Bridging this gap requires tools that demonstrate clinical value while fitting into existing workflows, which is essential to address ongoing implementation challenges, including usability, workflow integration, and cost-related concerns. This gap between technical feasibility and clinical usability underscores the need for AI solutions tailored to PHC’s specific workflow demands, resource constraints, and the effort required to transform routine practice.

PHC deals with broad, often undifferentiated presentations, requiring AI systems to handle multimodal data and variable clinical reasoning, unlike task-specific tools in specialized care. This challenge was evident in triage tools and symptom checkers, which performed inconsistently depending on use case and clinical context. These variabilities highlight the difficulty of designing AI systems that can replicate the nuanced and situation dependent–sensitive reasoning of GPs, which often relies on patient history, symptom presentation, and social context.

These challenges are compounded by broader system-level issues. Primary care providers worldwide face high levels of administrative burden and burnout, often driven by staffing shortages, complex EHR systems, and increasing time pressures. The COVID-19 pandemic further intensified these issues by accelerating the shift toward asynchronous, electronic, and nonvisit care models, while also fostering novel diagnostic pathways and forms of doctor-patient interaction [96]. In other sectors of health care, such as hospital administration, AI has already begun to ease such burdens through tools such as ambient digital scribes, suggesting that successful models for reducing workload exist but have yet to be fully adapted for PHC settings.

### The Human-Technology Divide in AI Adoption

A key theme emerging from the literature is the tension between the efficiency gains offered by AI and the central role of personal connection in PHC. Clinicians recognize AI’s potential to reduce administrative burden, a known contributor to burnout, and to enhance diagnostic precision. However, skepticism persists over issues of autonomy, interpretability, and transparency in decision-making. While AI tools for prescribing, risk assessment, and triage have demonstrated potential, hesitation persists around the risk of undermining clinical judgment and patient-centered care.

For patients, AI’s role in expediting referrals and diagnostic pathways was generally viewed positively, particularly when it improved access or screening uptake. However, a consistent preference for human-centered care and continuity in GP relationships emerged across studies. Given PHC’s emphasis on trust, shared decision-making, and holistic care, AI must be perceived as supportive of the clinician-patient relationship rather than replacing it. This suggests that AI systems designed to support clinical judgment, especially those developed through co-design with GPs and patients, are more likely to be accepted and integrated into primary care. The World Organization of Family Doctors’ Europe Future Plan 2023‐2027 identified delegable tasks as one of their thematic goals, in which AI can aid in improving GPs’ work [97].

### Equity and Global Challenges in AI Deployment

As seen in this review, the geographic concentration of AI research in high-income settings raises concerns about its global applicability. Tools developed in well-resourced systems may not perform reliably in low-resource environments, where infrastructure, data quality, and workflows differ significantly. Although the included studies represented several high-income countries, evidence from low-resource settings was limited. This geographic concentration raises concerns about the broader applicability of AI tools, especially in health care systems with different infrastructure, clinical workflows, or population health needs. Without validation in diverse contexts, AI systems risk introducing bias or failing to generalize across global primary care settings.

Given the concentration of included studies in high-income countries, inclusive AI development remains a priority. Ensuring equitable integration in primary care requires validation in diverse clinical and socioeconomic contexts. As PHC plays a critical role in promoting health equity, future AI tools should be developed with diverse data representation, bias mitigation strategies, and deployment models adapted to varied levels of health care access.

### Comparison With Existing Literature

Previous reviews have established AI’s emerging role in diagnostics, chronic disease monitoring, and administrative support, but gaps remain in understanding its practical implementation in PHC workflows. This review builds on earlier work by offering a broader perspective that contextualizes AI’s challenges and opportunities within real-world PHC settings.

A scoping review on AI use in PHC identified ML, natural language processing, and expert systems as the most commonly used AI interventions in community-based PHC, primarily for diagnosis, detection, and surveillance [18]. Our review corroborates these findings, demonstrating AI’s role in early diagnosis, decision support, and chronic disease management while also expanding the discussion to include operational efficiency and administrative automation.

In contrast to research which found that AI research in primary care is at an early stage and often lacks interdisciplinary collaboration and end user engagement, our study delves into the practical implications of AI integration within PHC, emphasizing its impact on clinical workflows and patient outcomes [17].

In other medical specialties, such as radiology and oncology, studies have similarly reported that despite promising technical developments, the real-world integration of AI tools remains limited. Common challenges across these fields include insufficient alignment with clinical workflows, limited trust in algorithmic outputs, unclear regulatory frameworks, and inadequate training for health care professionals. These issues closely resemble the barriers identified in our review of primary care, indicating that many of the obstacles to implementation are not unique to this setting. At the same time, the broader scope of patient presentations, the continuity of care, and the central role of the patient-clinician relationship in primary care may intensify these challenges. This comparison underscores the importance of developing AI implementation strategies that are not only technically robust but also sensitive to the everyday realities of general practice [98-100].

### Aligning AI with GP Roles

**Figure 2.**
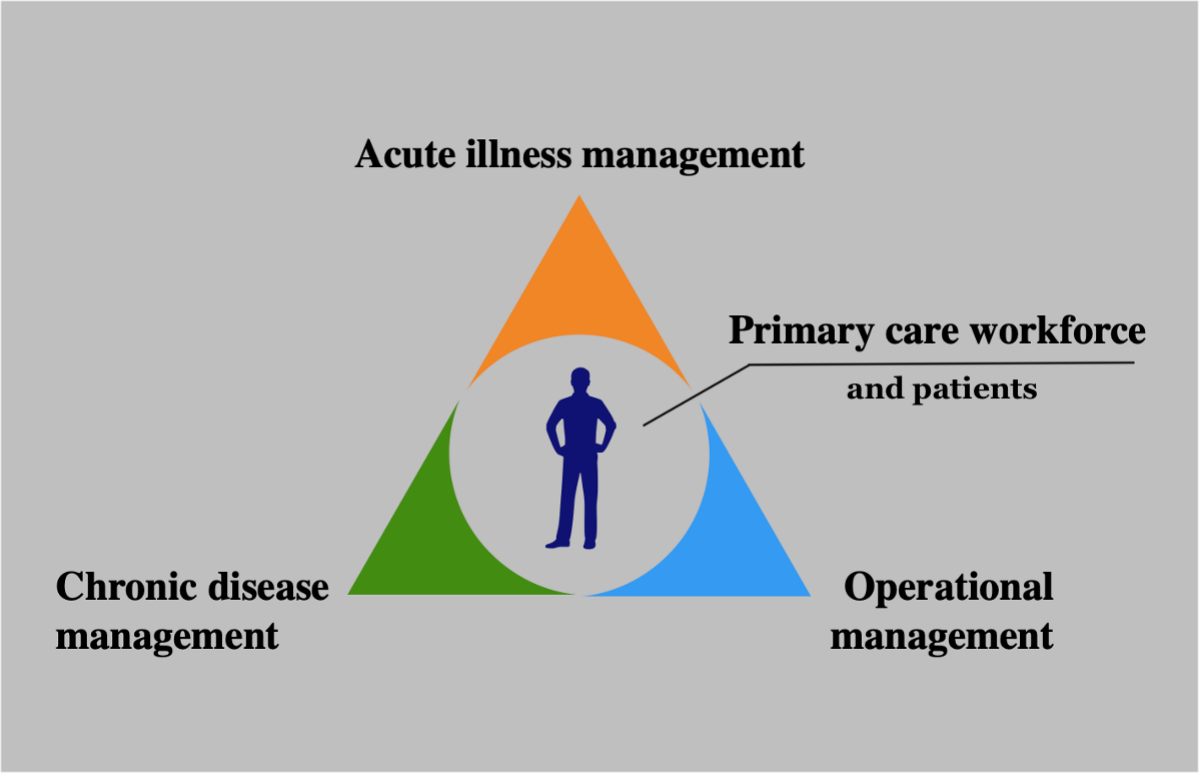
The triangle of PHC. A triangle diagram illustrating three core domains of primary care that have arisen during the thematic analysis: acute illness management (top), chronic disease management (bottom left), and operational management (bottom right). At the center of the triangle is a silhouette of a person, representing the primary care workforce and patients. This central figure emphasizes the human element at the intersection of all three management areas. PHC: primary health care.

Our findings can be conceptually mapped onto the fundamental roles of a GP. In this model, the physician is placed at the center of a triangle defined by acute care, chronic care, and practice management (Figure 2). The theme of early intervention and decision support directly enhances acute care by enabling faster, more accurate diagnoses and interventions during urgent encounters. Similarly, the theme of comprehensive chronic disease management supports the GP’s role in long-term patient monitoring and treatment adjustments, which is essential in managing chronic conditions. Lastly, the themes addressing primary care operations and user acceptance underscore the importance of effective practice management. This aligns with the distinctive characteristics of primary care data, which are often longitudinal, heterogeneous, and rooted in undifferentiated clinical presentations. These complexities demand tools that are not only accurate but contextually sensitive to PHC’s comprehensive scope [97].

### Limitations

This review has several limitations. First, this study was limited to 3 indexed databases and empirical, peer-reviewed research papers, potentially excluding relevant research from other databases or gray literature sources. The cutoff date of April 16, 2024, means that newer advancements, particularly in generative AI and evolving clinical applications, are not present.

Second, language bias is a limitation, as the review included only English-language publications, potentially omitting valuable research from non-English–speaking regions. Third, the included studies varied in design and scope, ranging from small-scale feasibility studies to retrospective analyses, making direct comparisons difficult; this is why we also refrained from critical appraisal.

Additionally, as a scoping review, this study aimed to map available literature rather than assess the quality or strength of evidence. Future systematic reviews with meta-analyses will be necessary to determine AI’s clinical effectiveness relative to standard care.

### Future Directions

To advance beyond narrow, disease-specific pilots, future research should adopt longitudinal, system-aware designs that reflect the real-world complexity of PHC. This includes evaluating how AI interacts with multimorbidity, time constraints, and relational continuity, elements that are often absent from current trials. Integrating patient experience and generalist clinical reasoning into evaluation frameworks will also be essential.

Beyond empirical research, the development of AI in primary care would benefit from structured, anticipatory planning. Future-oriented methods (such as scenario analysis and backcasting) can help stakeholders collaboratively envision pathways for responsible implementation. These approaches are well-suited to the uncertainties and ethical stakes of AI integration and offer a shared foundation for aligning innovation with the core values of primary care [101].

### Conclusions

This scoping review mapped the current landscape of AI applications in PHC, identifying tools aimed at early diagnosis, chronic disease management, operational support, and implementation experiences. While many tools demonstrated promising technical performance, especially those using structured clinical data, most of them were in an early testing phase and have not yet been integrated into routine practice. Common enablers across studies included alignment with existing workflows, structured data inputs, and clinician trust. However, persistent challenges, such as usability concerns, training gaps, and organizational barriers, continue to limit broader adoption. These findings emphasize that the future of AI in PHC depends not only on technological capability but also on thoughtful integration into the relational and practical realities of primary care.

## Supplementary material

10.2196/65950 Multimedia Appendix 1 Explanation of the search strategy.

10.2196/65950 Multimedia Appendix 2 The database used in the scoping review.

10.2196/65950 Checklist 1 The PRISMA-ScR checklist for this paper.

## Abbreviations

AI: artificial intelligence
ECG: electrocardiogram
EHR: electronic health record
GP: general practitioner
MeSH: Medical Subject Headings
PHC: primary health care
PRISMA-ScR: Preferred Reporting Items for Systematic Reviews and Meta-Analyses Extension for Scoping Reviews
