# Supplementary material for: AI and Primary Care: Scoping Review
Source: J Med Internet Res. 2025 Aug 15;27:e65950. doi: 10.2196/65950 (PMC12368388; doi:10.2196/65950)
Supplement: Multimedia Appendix 1 — Explanation of the search strategy. [file jmir-v27-e65950-s001.docx]

| PubMed |
| --- |
| (("artificial intelligence"[MeSH Terms] OR "machine learning"[MeSH Terms] OR "natural language processing"[MeSH Terms] OR "deep learning"[MeSH Terms] OR "artificial intelligence"[Title/Abstract] OR "machine learning"[Title/Abstract] OR "natural language processing"[Title/Abstract] OR "deep learning"[Title/Abstract]) AND ("primary health care"[MeSH Terms] OR "primary care"[Title/Abstract] OR "family practice"[MeSH Terms] OR "general practice"[MeSH Terms] OR "primary health care"[Title/Abstract] OR "family medicine"[Title/Abstract] OR "general practice"[Title/Abstract])) AND ("2001/01/01"[Date - Publication] : "2024/04/16"[Date - Publication]) |
| Scopus (articles were manually selected, if they were published before 2024/04/16) |
| (TITLE-ABS-KEY("artificial intelligence" OR "machine learning" OR "natural language processing" OR "deep learning"))  AND  (TITLE-ABS-KEY("primary care" OR "family practice" OR "general practice" OR "primary health care" OR "family medicine")) |
| Web Of Science (articles were manually selected, if they were published before 2024/04/16) |
| TS=("artificial intelligence" OR "machine learning" OR "natural language processing" OR "deep learning")  AND  TS=("primary care" OR "family practice" OR "general practice" OR "primary health care" OR "family medicine") |
